# Collaborative Creative Engagements as Drivers for Re-imagining Classrooms and Pedagogies

**DOI:** 10.1177/10778004241229063

**Published:** 2024-02-13

**Authors:** Natalie Tacuri, Mindy R. Carter, Layal Shuman, Daniel X. Harris, Christopher Blomkwist

**Affiliations:** 1 McGill University, Montréal, Quebec, Canada; 2 Toronto Metropolitan University, Ontario, Canada; 3 Royal Melbourne Institute of Technology University, Victoria, Australia

**Keywords:** creativity, pre-service teachers, participatory design, sketch modeling, Harris Creativity Audit

## Abstract

This paper presents a study examining how pre-service teachers understand and experience the limit(s) of classroom creativity in a Canadian higher education class. Participants first completed a modified version of the Harris Creativity Audit to assess their preliminary understandings of creativity policies and practices, as well as perceptions of the value and feasibility of incorporating creativity into their own teaching. The survey results informed the content of a two-part workshop where participants utilized participatory design and sketch modeling to further explore their understanding of classroom creativity. Data analysis resulted in three themes: (a) the impact of physical space; (b) assessing creativity and assessing in creative ways; (c) challenging the educational system. This study is part of a multisite Social Sciences and Humanities Research Council–funded project that aims to explore creativity in higher education to empower educators and students to develop creative agency through creative ecologies and collaborative assessment.

The research explored in this paper began when we, the co-authors, connected around the concern of creativity becoming a core competency in compulsory national curricula in Canada and Australia (Cachia & Ferrari, 2010; Heilmann & Korte, 2010). For example, the Canadian Council of Ministers of Education places creativity as a pan-Canadian global competency in which creativity plays an explicit role in preparing for future economic and social drivers. In Australia, the Australian Curriculum’s “critical and creative thinking” general capability assists students to build creative skills and capacities. Viewing creativity as a competency and means to further economic production on a large scale can be in tension with understandings of creativity as something that “displays [ . . . ] curiosity, collaboration, problem-posing, divergent thinking, persistence, innovation, mastery, productive risk-taking, synthesising, and critical thinking” (Harris, 2016, p. 43). This definition, centering our thinking, is a process of cultivation in which one needs to stop viewing creativity as a single input/output skill that can be easily transmitted, or simply a cognitive process separate from doing. Rather, creativity needs to be considered a complex habit of mind (Lucas, 2016; Nickerson & Sakamoto, 2010) that develops competency through a combination of “thinking” and “doing.”

While the arts have long advocated prioritizing creativity in education (Harris & Carter, 2021) as a way to develop a creative habit of mind, the question of how to move the development of creativity beyond the arts is a persistent one. Universities are well-positioned to develop more collaborative and creative learning environments as they provide a community context conducive to disrupting students’ knowledge paradigms and systems (Carter, 2014, 2022; Kwo et al., 2004; Shuman, 2018, 2022). For example, university students who have the opportunity to develop their creativity while working on collaborative projects may be more likely to transfer these creative abilities and understandings into their future jobs and lives (Carter, 2010; Harris, 2016; Howard et al., 2018; Shuman, 2018).

In this paper, we explore students’ understanding of their creativity using qualitative research methods including a survey, a two-part participatory design workshop, focus groups, and an interview. Participatory design can be understood as an umbrella term for the different types of design approaches that actively involve the people being affected by a particular issue to collectively develop solutions for the purpose of meaningful action and change (Vaughn & Jacquez, 2020). Although scholars have documented challenges to using a participatory design (i.e., lack of resources, unpredictability, time, and so on) in the research process (Kujala, 2003; Steen et al., 2011), collective engagement between researchers and communities has been proven to allow for better-quality solutions that are more responsive to the needs of the affected population (Carter et al., 2021; Hoyer et al., 2010; Kujala, 2003; Steen et al., 2011), have higher rates of adoption for the solutions (Nyonka & Yoo, 2016), and offer opportunities to strengthen agency and build confidence in its users (MIT D-Lab, n.d.).

We examined pre-service teachers’ understandings of learning ecosystems by drawing upon a modified version of the Harris Creativity Audit and Creative Ecologies Model (Harris 2014; 2016). Harris’ Creativity Audit allowed for us to move toward an understanding of how learning ecosystems promote collaborative and transdisciplinary teaching and learning experiences that foster creativity.

## Harris Creativity Audit

**Figure 1.**
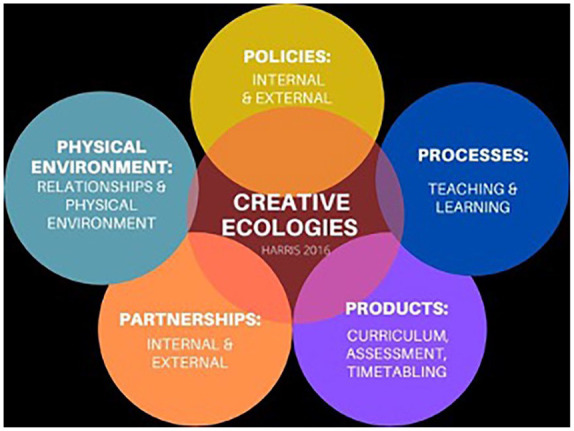
Creative Ecologies Model (Harris, 2016).

The Harris Creativity Audit and Creative Ecologies Model (Harris, 2016) is a suite of tools for assisting organizations and organizational units to increase their creative capacity using a holistic approach rather than a more individual, humanist, and largely cognitive (“talent”) approach. Research has shown the ways in which creativity is fostered not only by nurturing environments but also by a range of socio-cultural factors including time, context, collaboration, safety, and outcomes required (Kaufman & Glăveanu, 2021). The Harris Creativity Audit (Figure 1) uses the five domains of Processes, Products, Partnerships, Place (Environment), and Policies to review these interconnected axes of creative possibility. It also highlights their interdependence to demonstrate the ways creativity never emerges from only one single source or stream but simultaneously from the weaving of them all. Unlike other creativity heuristics (think the “4 C’s” and so on), the creative ecologies approach is not simply naming important aspects of creativity, but rather focuses on the ecological or interconnected nature of the five domains (Kaufman & Beghetto, 2009). The Harris Creativity Audit breaks out a set of questions/provocations to be used for assessing the creative capacity of each of the five components of any ecology—from classroom, to faculty, to business unit, to research team, to city-site. The creative ecologies model and audit can be used for any group to review its creative capacity (see Harris, 2016, pp. 37–42).

## Methods

We worked with 43 undergraduate students from a first-year Communication in Education course from a Canadian University’s Bachelor of Education program. We adapted Harris’ Creativity Audit (Harris, 2016) as a survey, conducted a two-part participatory design workshop, and ran follow-up focus groups and an interview to explore students’ understandings of creativity and their readiness to engage in creative pursuits in a collaborative classroom context.

### Participants

The participants in this study were recruited from a Communication in Education course. Prior to data collection, two virtual meetings were held to introduce the potential participants to the research project’s objectives and to get to know the researchers and participatory design facilitator who collaborated with us during this part of the project. Due to the state of the COVID-19 pandemic in February 2022, these recruitment meetings were held virtually via Zoom. A total of 40 pre-service teachers participated in the survey as a part of their class. While all 43 pre-service teachers in the class participated in the participatory design workshop, six students ultimately participated in the focus groups, and one in an interview.

### Data Collection

#### Survey

**Figure 2.**
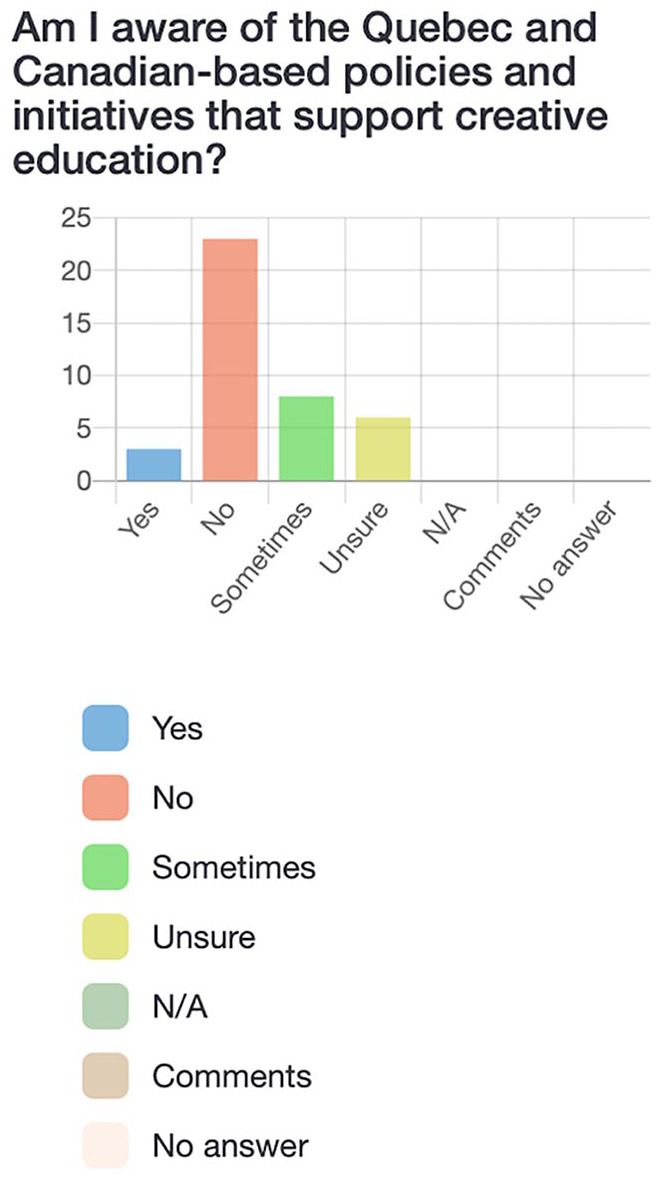
Participants’ responses to survey question about awareness of creative education policies and initiatives.

**Figure 3.**
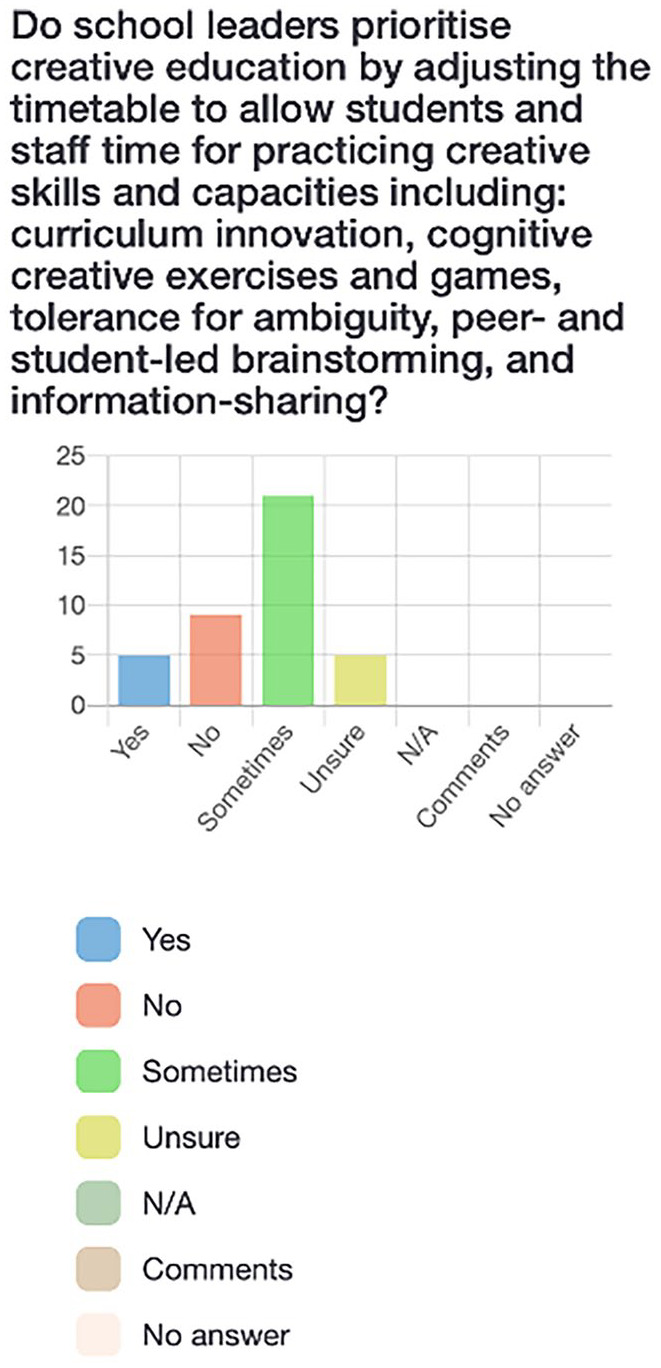
Participants’ responses to survey question about school leaders prioritizing creative education.

**Figure 4.**
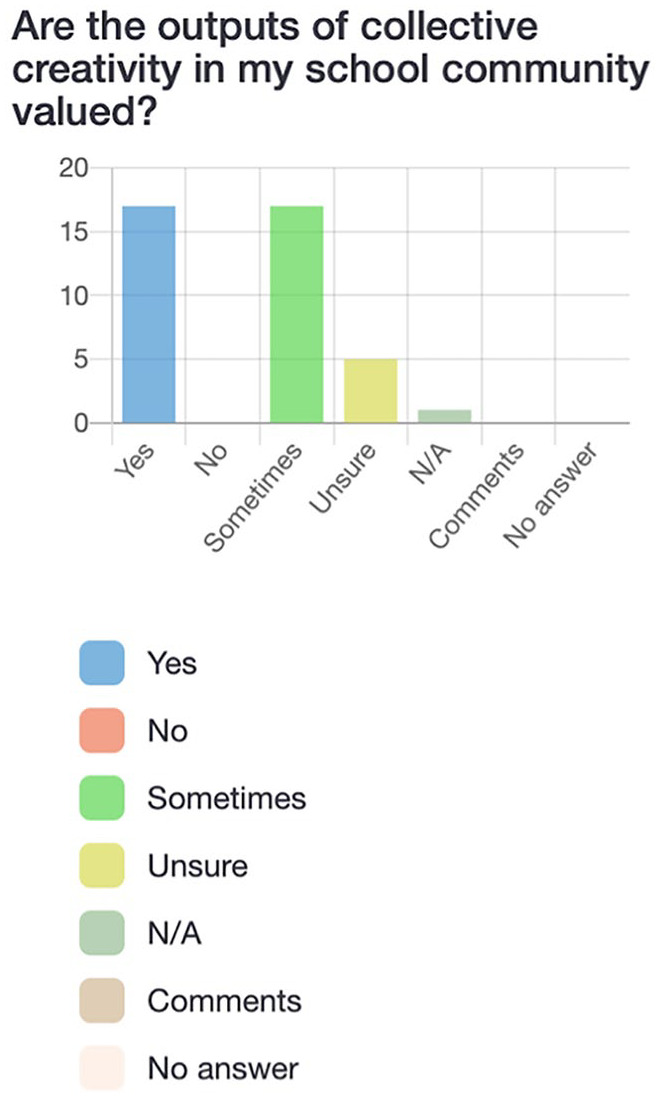
Participants’ responses to survey question about the value of creative outputs.

**Figure 5.**
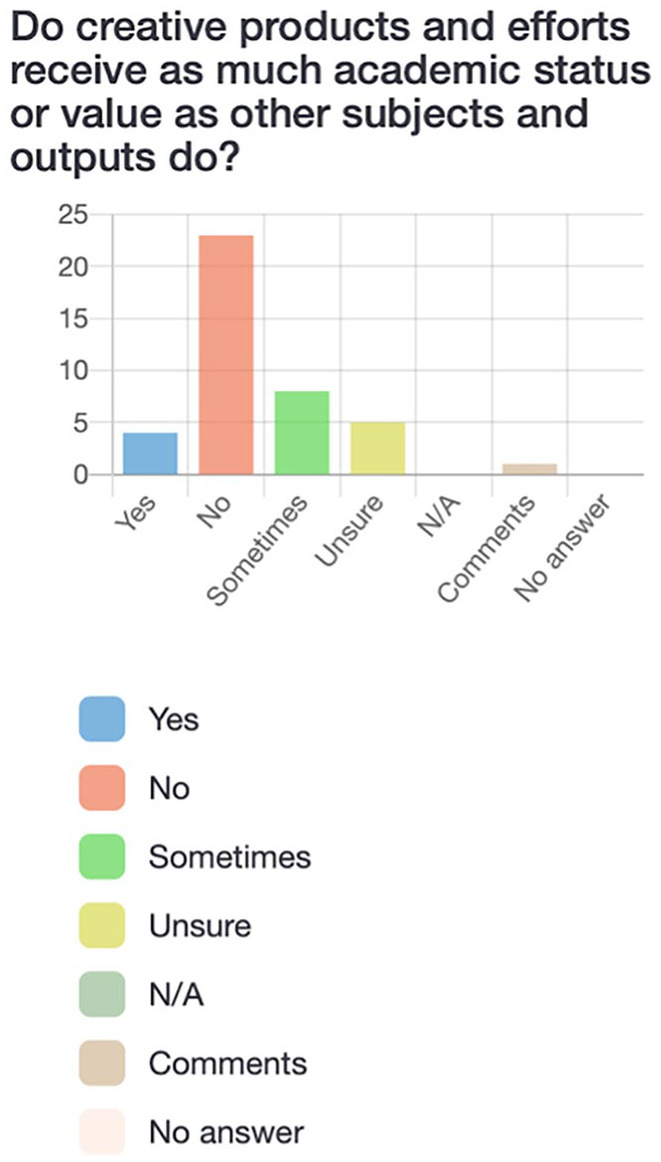
Participants responses to survey question about the value of creative products and efforts.

Participants first completed a survey via LimeSurvey, an online platform. The survey consisted of 21 questions (see Figures 2–5 for examples) which allowed participants to respond “yes,” “no,” “sometimes,” “unsure,” or “not applicable” and provided the option to leave a comment for each question. Participants were asked questions such as: “Am I aware of the Quebec and Canadian-based policies and initiatives that support creative education?” and “Do I have opportunities (and an obligation) to practice creative thinking, doing, and sharing in school?” The purpose of the survey was to assess pre-service teachers’ preliminary understandings of creativity policies and practices as well as individual perceptions of the value and feasibility of incorporating creativity into their own teaching.

#### Participatory Design Workshop

Once the survey was completed, these results informed the content of a subsequent two-part workshop in which participants utilized participatory design and sketch modeling to further explore their own understandings of creative ecologies. The workshop moved participants away from examining individual creativity and instead focused on how considering an educational issue that they wished to problematize collectively could be understood more deeply through a prototyping process. The workshop days were held in-person three weeks apart as COVID-19 restrictions began to ease. The first workshop day began with leading participants through a series of icebreaker activities, which were a key component of the workshop as they helped to facilitate a collaborative, playful, and creative environment for researchers and participants. After the icebreakers, we reviewed the results of the survey where participants’ responses indicated they generally valued creativity but demonstrated tension in understanding how creativity was situated within policy documents and within the curriculum.

**Figure 6:**
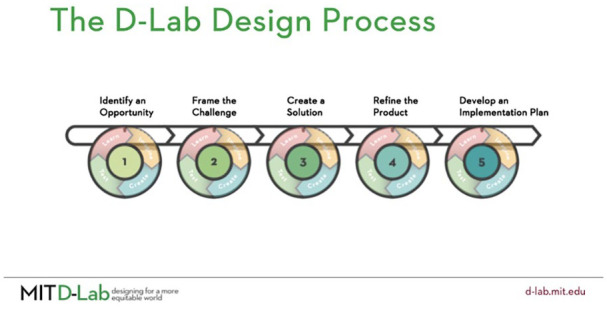
The Participatory Design Process.

Participants were then introduced to the participatory design process (see Figure 6) and discussed how this approach could help support their collective values surrounding creative ecologies. In small groups, participants had the opportunity to work through the first two stages of this process by responding to the following question: “How might we improve opportunities to practice creativity?” In doing so, we asked participants to collectively decide upon the challenge they felt was most urgent to them as pre-service teachers that could be further explored in the next workshop. Participants began preliminary discussions about problems pertaining to time and resources for creativity in the classroom, removing the focus on grades, rearranging classroom spaces, and negotiating power dynamics between teachers and students.

On the second workshop day, participants worked through the third stage of the participatory design process using sketching and modeling. We gave participants a variety of materials (i.e., clay, pipe cleaner, cardboard) to visually present their emerging solution. Visualization was used as it aligns well with the participatory design process in accommodating a variety of communication styles, connecting to different types of intelligences, democratizing the design process, and reducing power dynamics (Kujala, 2003). Toward the end of the workshop, participants had the opportunity to describe their models to the class, provide feedback to their peers, and continue discussions on their creative solutions for improving opportunities to practice creativity in the classroom.

#### Focus Groups and Interview

We invited participants to participate in follow-up focus groups or interviews to reflect on their experiences in the workshop, describe their previous experiences with classroom creativity, and discuss their willingness to teach and assess creativity as pre-service teachers. Two focus groups were conducted virtually three weeks after the final workshop day via Zoom. To accommodate schedules, one participant opted to participate in a one-on-one interview, answering the same questions as the focus group participants.

### Data Analysis

We analyzed the results of the survey using the statistics feature on LimeSurvey to visually display results for each question. As these preliminary results were used only to inform the content of the workshop, no advanced statistical analyses were conducted. The workshop, focus groups, and the interview were transcribed using NVivo transcription and were checked for accuracy by a research assistant. Following transcription, two members of our research team met virtually via Zoom to begin a coarse-grained analysis (Butler-Kisber, 2010; Carter, 2022) in which we reviewed the transcripts, discussed broad categorizations of the data, and began conceptualizing preliminary themes. Upon agreeing to three preliminary themes, the principal investigator conducted a fine-grained analysis (Butler-Kisber, 2010) on the focus group and interview transcripts while the research assistant analyzed the workshop transcripts in each of the major themes. A second meeting was then held to discuss which portions of the transcripts were placed into each theme, refine the names of each theme, and clarify rules for inclusion (Butler-Kisber, 2010). In this meeting, the two researchers worked back and forth to finalize their thematic categorizations of the data.

## Results

The results of the survey, workshop, focus groups, and interview led us to three themes to exemplify how participants understood and experienced the limits of classroom creativity in higher education: (a) the impact of physical space; (b) assessing creativity and assessing in creative ways; and (c) challenging the educational system.

### The Impact of Physical Space

In this first theme, participants challenged the notion of the “traditional” classroom and discussed its limitations to fostering creativity for both students and teachers. One workshop participant described her group’s perspective as pre-service teachers: “We were working from the assumption that yes, as teachers, we value creativity. So it’s not so much about fostering that as much as it is implementing it in the space itself.” Participants explicitly stated they valued creativity in the workshop, and through their survey responses, they further pointed out a collective need to adapt the classroom space to reflect this value. They described university classrooms, specifically, as being “just a brick wall” that is “cramped with not enough space.” One workshop participant questioned, “how do we implement these ideals in classrooms, which are often packed with students and might not necessarily have the space to have distinct desks over here, activities over here?” Another workshop participant elaborated on these physical limitations:

How might we arrange classroom spaces to promote creativity? So this came just from looking around this room, the walls are bare, there are no windows and it’s awful lighting, and that’s not conducive to creativity for us as adults. I can’t even imagine for younger students . . . So that comes down to just the way the seats are arranged. Can we put things onto the walls that either have quotes or even colours, something that really can speak to the crowd and allow that creativity to take over? . . . Can there be a reading corner? Can there be this kind of corner? Just different stations, maybe to allow students to go and express themselves and be creative freely.

Contrastingly, two focus-group participants noted positive experiences with fewer physical constraints in the university context. They explained, “it would have a circular layout so that everyone was facing each other and it was a bit more conducive to discussing” and “classrooms that were laid out differently and a bit more democratically, I noticed it made a real difference in discussions.” When comparing the rigidity of traditional classroom spaces to the openness created by alternative classroom setups, as exemplified in the aforementioned quotes, participants began to think through tangible solutions to the classrooms they had experienced thus far. Participants offered creative suggestions including painting walls, implementing a reading and/or activity corner, bringing in greenery, and bringing in miscellaneous items (i.e., candles, lamps) to make the classroom “a comfortable space for students, but also for teachers” (Workshop Participant). One workshop group also considered accessibility when brainstorming potential solutions:

How can we adapt the classroom setting to promote creativity? Our group discussed the ways we could move desks or do the students even have to be sitting throughout the class period? Because it’s been shown that getting up can be more productive and probably more stimulating for students and in turn, more accessible to students who maybe have ADHD or something like that will promote their learning.

**Figure 7.**
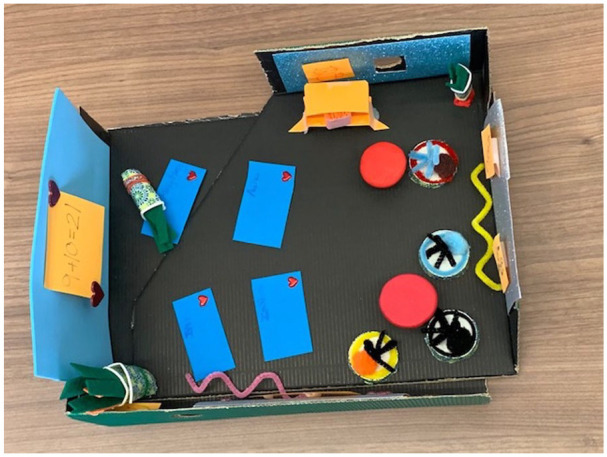
Sketch Model of Alternative Classroom Arrangement.

Accessibility was discussed by only one workshop group, while the most common solution discussed by participants pertained to alternative seating arrangements and collaborative spaces. Three workshop groups focused their participatory design processes on these concepts. When discussing their sketch model (see Figure 7), a participant in one of these groups expressed:

As we were building our mini classroom, one of the ways we approached that is dividing the space up so that there is the more traditional lecture hall towards the front of the classroom. But there’s also collaborative group spaces and a reading area . . . and have distinct areas where you can separate sitting down and just listening to a lecture style class and then actually having the space to move around and look at each other and talk to each other and create together.

Altogether, the first theme emphasized participants’ awareness of the potential of the physical classroom environment to either support or inhibit creativity for teachers and students. In thinking through ways to challenge traditional norms in a physical sense, participants also began to work through various ways to challenge the rigidity of assessments and grading.

### Assessing Creativity and Assessing in Creative Ways

When discussing assessments, participants grappled with challenges pertaining to assessing creativity and assessing in creative ways. From the perspective of the teacher, participants noted that teachers may feel apprehensive to take risks in the way that they grade, may not know how to develop creativity outside of a subjective evaluation of a students’ effort, and may not understand how much creative agency they have within the boundaries of the curriculum and the educational system more generally. In one of the focus groups, a participant expressed:

Sometimes teachers are scared to take the risk and it’s hard to know how to grade creativity. I feel like in my experience, all I’ve been graded on for creative assignments was effort. And that feels problematic sometimes because how can you tell me that my effort was more or less than [another student’s] effort?

This same participant referenced the influence of the educational system in pushing teachers toward producing a grade that is recognized by the curriculum. Others in the focus group reflected these ideas by discussing the challenges of assessing creativity in a way that could be validated and recognized by larger governing bodies in education. Not only was this difficult for teachers to accomplish within the constraints of the educational system, they also acknowledged that students may not be receptive to being graded on their own creativity and in creative ways. One workshop participant asked, “how can you convince students who would rather have a concrete numerical grade that a visual representation is more effective?”

In reflecting on their own assessment practices, participants were generally open to the idea of challenging the “concrete numerical grade” to “encourage more creative freedom.” Many participants referenced their prior experiences with “strict rubrics in the classroom” and the ways in which these prior experiences drove them to find flexibility within a seemingly rigid structure. When discussing rubrics, one workshop participant noted, “if you think about them, it’s always these boxes. If you’re always trying to fit into that, the five on five box. And so that kind of limits how far you can go as far as creativity.” Similarly, another workshop participant expressed, “this boxy rubric idea that really restricts students’ ability to step outside of the box because there’s literally a physical box that they’re looking at that they have to be in order to reach that, let’s say that five on five.”

**Figure 8:**
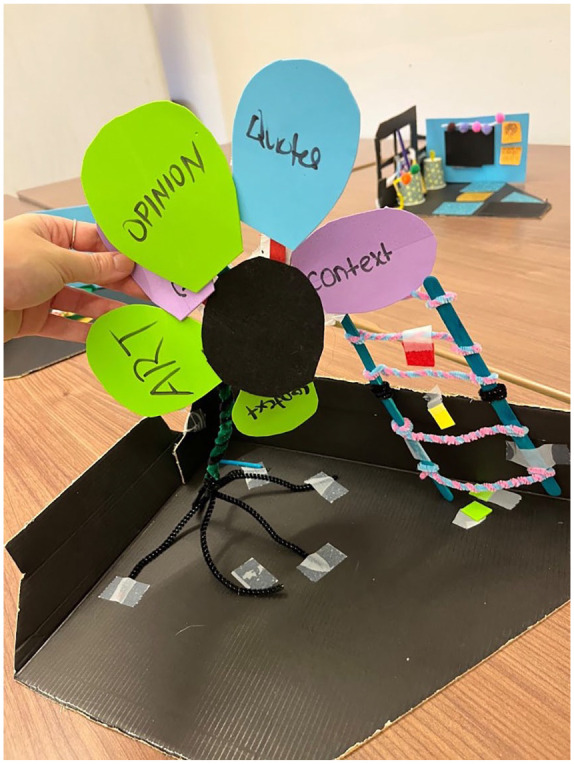
Sketch Model of Alternative “Flower Rubric”.

To address these limitations, one workshop participant pointed to the overall possibility of “allowing space for students to be able to do things that are different. So not having students do the same things and grading them on that aspect [doing things differently].” Another workshop participant elaborated, “given that we all grew up on very strict [guidelines], you have to write this paper, this is the layout and everything, and there was never any creativity in that. So we want to change that.” Two groups of workshop participants focused their problem framings on addressing these challenges. Notably, one of these groups created their sketch model in the form of a flower (see Figure 8) to address the limits of creativity for teachers when assessing students and for students when completing course-based assignments.

One workshop group member explained:

At the root of the flower, you have like all your prior knowledge. So the things you learn in class, the things your teacher tells you, like student ideas and stuff like that. Then afterwards, your stem is kind of the purpose of your assignment, what you want students to get out of this assignment. And then each petal is kind of like something students can do in order to fulfill the purpose of this assignment . . .. And in the end, [students] kind of come up with something that they created on their own and it kind of allows them to be creative in a way.

This particular group used their alternative flower rubric to maximize autonomy in their students. They expressed this as important to them as pre-service teachers in stating:

For us, the most important thing was just to remove keen focus on grades and to really allow students to be creative in their own way, in a way that’s not the teacher telling them, oh, you have to do these three things in order to have an A. It’s removing that idea, just making it more open to discussion.

The incorporation of the ladder in Figure 6 was described by participants in this group as a visualization of their students “hitting objectives” that they set out for themselves, rather than specific tasks set out by the teachers.

This “flower rubric” was one of many ways participants discussed promoting flexibility in evaluations and assessments. However, in each of the solutions presented, participants were faced with a consideration of various institutional barriers that hindered their creative choices and pushed them to reconsider the feasibility of their solutions. In line with assessing creativity and assessing in creative ways, the last theme discovered was challenging the educational system.

### Challenging the Educational System

In this final theme, participants thought more broadly about creative ecosystems to reimagine the institutional structures they felt were a hindrance on their own creativity as pre-service teachers. Various participants spoke broadly about “norms” and “traditions” in teaching practices and the ways in which there seemed to be an “unwillingness to stray from the normal and the traditional and not [take] the time to learn new things and try and understand the newer ways” (Workshop Participant). One workshop participant re-framed this “unwillingness” by saying:

Sometimes we forget that the teacher is part of the system. And so the criteria of creativity sometimes might go against the criteria of the whole system. And so how does a teacher navigate these differences? Because sometimes the teachers want to provide more options, but they can’t because the system doesn’t allow it.

When discussing challenges for incorporating creativity within the educational system, time was a major point of reference for participants. One workshop participant stated:

A lot of creative work does take time . . . for some people, they can just sit down and knock it out. But for a lot of students, it’s a much lengthier process that involves more steps or involves more time to just kind of sit . . . which you don’t always have time for as a teacher.

This participant elaborated on the challenge of time by expressing:

If you only have a fifteen-minute block or you have so many things within the curriculum that you have to knock out before the end of the semester, you don’t have the time to kind of carve that space out in the classroom, and you can’t always expect your students to be able to carve that space out.

Another participant in her workshop group suggested “compress[ing] the curriculum” to allow more time for other creative pursuits in the classroom. It was important for this group to find time within the school day for creativity rather than asking students to work creatively at home, as they considered students’ part-time jobs and family responsibilities outside of the classroom.

Nevertheless, a focus group participant cautioned this “compressing” of the curriculum by stating, “my concerns are more of what I’m allowed to do in the curriculum or what I’m allowed to do in the classroom in terms of what I have authority over.” Various participants echoed this concern, as they, too, felt that they struggled to understand the level of creative agency they had in the classroom while maintaining their teaching of the curriculum. A focus group participant referenced an overall need for “more policy literacy” to assist their understandings of “what is flexible, what is rigid” and how much autonomy they have within the limitations placed on them. When reflecting on the knowledge gained from the workshop, an interview participant shared similar feelings:

I think one of the major takeaways is that a lot of people . . . didn’t really know a lot about the creativity things going on in like . . . the QEP or whatever. Or there are some things in the curriculum of schools and how the government is implementing this . . . I really had no idea that this was happening.

Most participants reflected on the challenge of incorporating creativity within the curriculum as a concern for teachers who had little to no support in understanding creative policies and guidelines. One workshop participant shifted the conversation in pointing to students as potential supporters, “I feel like a lot of times you miss an entire resource, which is the students themselves.” She discussed the difficulty in working within a “very adultist society” where the teacher is the “master in the classroom.” Other participants expanded on the conversation surrounding teacher-student power dynamics where the teacher is the ultimate authority figure, particularly male teachers with female students, as one workshop participant felt that “the male teacher might not be willing to listen to female students, and that might discourage them from expressing their creativity to the full extent.”

**Figure 9.**
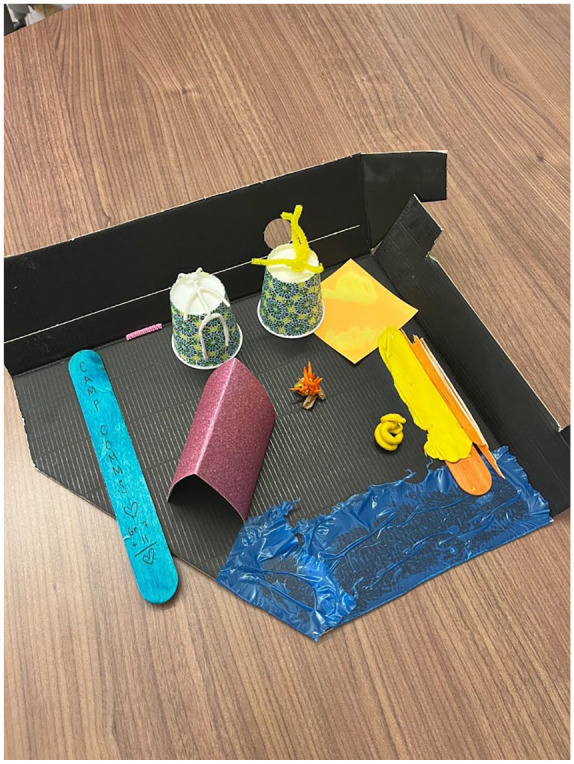
Sketch Model of Peer Advising Program.

Notably, one group focused their problem framing and sketch model (see Figure 9) on power dynamics between students themselves rather than teachers and students. This group acknowledged the impact of the educational system in creating hierarchies between students, specifically between students of different ages. Through the conversations involved in creating their sketch model, this group proposed the potential of a peer advising program where older students are matched with younger students to provide opportunities for mentorship. The group discussed the benefits of this program as it could instill:

more confidence in both the younger and the older students based on . . . the younger kids get to know the older kids and that’s very cool, as well as the older kids get to be a leader for someone. So that builds confidence. And then through confidence, there’s also a sense of community that comes through the peer advising program, which then encourages creativity.

The implementation of a peer advising program was one tangible solution created by participants in pushing back against the challenges of the educational system. However, participants struggled with finding solutions to the aforementioned difficulties surrounding time, working creatively in conjunction with teaching the curriculum, and working through teacher-student power dynamics. The restrictive environment created by the educational system was described by a focus group participant as “foster[ing] a certain kind of learning for a certain kind of outcome . . . So we’re not really thinking broadly of what creativity and learning are. It’s still within the bounds of what the institution wants.” Overall, these barriers remained a space where participants felt bound by the institution and left feeling unsupported by the government and policy-makers.

## Discussion

In this study, we used the Harris Creativity Audit (Harris, 2016) as a framework to understand students’ experiences of creativity in pre-service teacher education. The framework invites us to consider a holistic ecological approach when evaluating creativity and how aspects like learning environments, pedagogies, students’ and educators’ creative practices, and external partnerships cultivate creativity within educational settings. The three findings that we generated include (a) the impact of physical space, (b) assessing creativity and assessing in creative ways, and (c) challenging the educational system. The findings echo others of studies such as Cachia and Ferrari (2010) and Glăveanu et al. (2020), as well as Harris and de Bruin (2018, de Bruin & Harris, 2017) who move us beyond fixating on individuals’ characteristics and capabilities when studying creativity education. The study also reveals the lack of defined pedagogical methods, educational approaches, and structures that support creativity in pre-service teacher education despite its increasing interest. When pre-service teachers are not consistently learning throughout their program about creativity through assignments, assessments, and creative cultures that they experience around them, as our research participants shared, they feel ill-prepared to teach creativity to their students in the future. Harris and de Bruin (2018) connected the vagueness around creativity in education with more significant implications, arguing that “while creativity and critical thinking play an explicit role in developing and understanding future economic and social drivers, notions of what creativity is in an educational sense remain vague to both policymakers and educators” (p. 154). In other words, while the interest in teaching people to be creative exists, the translation of this interest is yet to be materialized across curricula and educational settings that hope to scaffold students’ creativity. In what follows, we discuss the three findings as they relate to broader studies and how they contribute to our understanding of creativity in pre-service teacher education specifically but could also hint at the state of creativity education in undergraduate programs in higher education.

Research participants emphasized how physical spaces impact creativity education in the first finding of our study, the impact of physical space. With large lecture-style classrooms and concrete walls around us, we often forget how “uninspiring” it is to engage in creative thinking and practices within our university spaces. Students in the study reminded us of this phenomenon by describing their classrooms as “rigid” and “traditional.” They shared how these physical environments hinder their excitement to pursue creative endeavors. Research participants also designed prototypes of spaces that they deemed inspiring for creativity. The outcomes were maquettes of classrooms they believed could stimulate creativity. Their features included colorful walls, various prompts inviting creative explorations, and open space layouts with corners for activities such as reading and crafts. Physical space may seem a minor aspect of fostering creativity in formal educational settings, but similar to research participants, numerous creativity scholars such as Beattie (2000) have emphasized the influence of physical space on creativity teaching and learning. With the corporatization of higher education resulting in an increasing number of students in large lecture halls, educators hoping to foster creativity will continue to be exhausted by the difficulties these spaces present for them to nurture relational, artful, hands-on, and overall, creative pedagogies, as our first finding highlights.

In the second research finding, assessing creativity and assessing in creative ways, research participants described their anxieties about assessing creativity. They felt ill-prepared to shift from traditional assessment modalities and worried that experimenting with methods for creativity assessment would negatively affect their relationships with their students. They also were not sure their students would be comfortable being assessed on their creative abilities. These beliefs demonstrate the prevailing ambiguities in teaching and learning creativity. Pre-service teachers feel mal-equipped to create assessment methods that support their creative assignments and fear backlash from students. This finding contributes to many scholarly conversations about the need to demystify assessment practices for creativity while acknowledging that creative assessment methods require effort but can be learned and developed (Beattie, 2000; Cachia & Ferrari, 2010; de Bruin & Harris, 2017; Harris et al., 2022).

The third finding, challenging the educational system, emphasizes building educational systems that support creativity. Research participants described their institutional structures as hindering their creativity. They felt that educational policies and guidelines were disconnected from their day-to-day learning as pre-service teachers. Participants also believed the current educational system breeds adverse outcomes, such as creating hierarchies among students and uneven power dynamics between teachers and students. Like participants in the study, many researchers warned us that for creativity education to flourish, the larger systems and structures that carry the pedagogies and learnings of teachers and students need to support and not obstruct their creativity. Because we examined students’ experiences through the Harris Creativity Audit framework (Harris, 2016), we could see the missing components described in the study and imagine an ecology that would enable students to thrive creatively. The framework allowed us, more so, to evaluate creativity from our research participants’ perspectives beyond them as individuals and their capabilities to be creative. Similarly, the finding builds on the work of many others who explained that we should investigate the environments within which creativity may or may not occur and take on an ecological perspective when researching creativity education, thus moving us away from singular or incidental analyses of teaching and learning creativity toward more holistic and ecological approaches.

## Conclusion

We have presented a study exploring pre-service teachers’ experiences and understanding of creativity in the classroom in a Canadian higher-education class. A main driving factor behind our research is that creativity is becoming a core competency in national curricula in Canada. We utilized the Harris Creativity Audit (Harris, 2016) as a framework to examine these experiences and beliefs. Harris’ framework promotes an ecological approach when evaluating creativity and how aspects like learning environments, pedagogies, students’ and educators’ creative practices, and external partnerships cultivate creativity within educational settings.

Forty-three participants, all pre-service teachers, were first asked to fill out a modified version of the Harris Creativity Audit to determine their initial beliefs and awareness of creativity policies, practices, and perceptions of the value and feasibility of incorporating creativity into their teaching. These survey results were used to direct a two-part workshop where participants utilized participatory design and sketch modeling to continue to explore their understanding of classroom creativity. Follow-up focus groups and an interview were also conducted to examine their understandings of creativity and their eagerness to engage in creative endeavors in a collaborative classroom context. Through completion of our study, three findings were generated: (a) the impact of physical space, (b) assessing creativity and assessing in creative ways, and (c) challenging the educational system.

The students’ perspectives of the impact of physical space was concerned not about their valuing of creativity but about the need to adapt the classroom space to express this value. Students felt that traditional classrooms were nothing more than “just a brick wall.” For creativity to blossom, classrooms should be designed to encourage creative thoughts, with suggestions including alternative seating arrangements and collaborative spaces. One group also noted the importance of accessibility to the classroom.

When considering assessing creativity and assessing in creative ways, students felt that teachers may be apprehensive about taking risks in how they grade, may be unsure how to establish creativity outside of a subjective evaluation of a student’s effort, and may not understand the level of creative agency they have within the confines of the curriculum and the educational system.

Finally, when exploring students’ perceptions of challenging the educational system, many participants discussed the creative ecosystem as a whole. Numerous students spoke about the “norms” and “traditions” within educational systems and the perceived lack of self-efficacy of teachers to incorporate creativity in their assessment. Furthermore, some participants asserted that time was also a factor in the lack of creativity in the classroom.

Overall, this study encourages us to depart from focusing on individual characteristics and abilities when studying creativity education. When students are not persistently learning about creativity through assignments, assessments, and creative culture within the classroom, they feel poorly prepared to teach creativity when entering the educational workforce.

Finally and most importantly, the study also unveils the absence of defined pedagogical methods, educational approaches, and structures that assist creativity in pre-service teacher education, regardless of its ever-increasing interest and importance. While there is interest in teaching people to be creative, this interest has yet to emerge across curricula and in educational settings that build up students’ creativity.
